# The Link between Attachment and Gambling in Adolescence: A Multiple Mediation Analysis with Developmental Perspective, Theory of Mind (Friend) and Adaptive Response

**DOI:** 10.3390/jpm11030228

**Published:** 2021-03-22

**Authors:** Grazia Terrone, Alessio Gori, Eleonora Topino, Alessandro Musetti, Alessia Scarinci, Camilla Guccione, Vincenzo Caretti

**Affiliations:** 1 Department of History, Cultural Heritage, Education and Society, University of Rome Tor Vergata, 00133 Rome, Italy; 2 Department of Human Sciences, University of Florence, 50100 Florence, Italy; gori.alessio@gmail.com; 3 Department of Human Sciences, Lumsa University of Rome, 00185 Rome, Italy; eleonora.topino@gmail.com (E.T.); c.guccione@lumsa.it (C.G.); vincenzocaretti@gmail.com (V.C.); 4 Department of Humanities, Social Sciences and Cultural Industries, University of Parma, 43121 Parma, Italy; alessandro.musetti@unipr.it; 5 Department of Education Sciences, Psychology, Communication, University of Bari, 70121 Bari, Italy; alessia.scarinci@uniba.it

**Keywords:** gambling disorder, attachment, adolescence, friend and family interview

## Abstract

**Introduction**: Several studies have supported the evidence that attachment styles are a central factor in adolescent gambling problems. On this theoretical basis, the aim of the present study is to analyze a hypothesized mediation model exploring both the direct and indirect effects of insecure attachment on gambling disorder by investigating the role of the developmental perspective, theory of mind (friend) and adaptive response in that relationship. **Method**: The sample consists of 178 adolescents who underwent the *Measures: South Oaks Gambling Screen—Revised for Adolescents* and *Friends and Family Interview*. **Result**: The mediation analysis was conducted following Hayes’ (2018) procedure, using Model 6. The results showed a significant association between insecure attachment and gambling disorder (*β* = 0.669; *p* < 0.001). The findings also highlighted a significant chained mediation model in which insecure attachment negatively influenced the developmental perspective (*β* = −0.742; *p* < 0.001), which affected the theory of mind toward one’s own best friend (*β* = 0.352; *p* < 0.001). **Conclusions**: The results highlighted a significant role of insecure attachment in predicting the symptomatic expression of gambling among adolescents, specifically impacting the development perspective, theory of mind toward one’s best friend and adaptive response to stress, which were linked to each other by a sequential influence. Therefore, our results showed that a poor developmental self-vision predicted a dysfunctional theory of mind toward the best friend. This could hinder the formation of positive peer relationships, which are crucial for the development of one’s identity.

## 1. Introduction

Research has shown that gambling is a popular conduct among adolescents, with high rates of problematic and pathological gambling [1]; they can indulge in classic and popular types of gambling, but authors have highlighted a steady increase in novel forms of gambling via the Internet [2,3,4], with greater local availability and accessibility [5]. Adolescent gambling may lead to negative consequences such as problematic relationships, delinquent and aggressive behavior [6], depression symptoms [7], increased risk of attempted suicide, increased risk of comorbidity with other forms of addiction [8] and general health problems [9,10,11,12].

Several studies have supported the evidence that attachment styles could play a key role in adolescent gambling problems [13,14,15,16]. Indeed, a growing body of research analyzed the relationship between adolescent gambling and attachment styles and found a higher incidence of insecure relationships with caregiver in gamblers and also links between alexithymia, attachment, and gambling disorder [17,18,19]. More specifically, insecure attachment hinders the development of adequate regulation skills and this predisposes one to emotional maladjustment [20]; therefore, addictive behaviors can be seen as an attachment disorder [15,21,22] and as an attempt at self-medication [23,24,25]. Indeed, previous research showed that gambling behaviours may act as external regulators of internal emotional states [17,26,27,28] and insecure attachment could be a vulnerability factor for its onset [29,30].

According to this framework, the aim of this present study was to investigate the impact of insecure attachment on gambling disorders in adolescence and to analyze the mediating role of several related variables. Indeed, the internal working models modeled in early childhood will influence aspects that are still being defined in this delicate and important life stage, such as the temporal perspective [31], the quality of relationships with peers [32] and the ability to provide adaptive responses to distress [33].

During adolescence, one acquires a greater awareness of his or her identity, taking up and creating his or her own memories of the past. At the same time, greater importance is put on the future, including the realization of one’s aspirations and projects [34,35,36]. This developmental self-vision, which is linked to one’s entire past, present and future axes, is extremely influenced by relationships with one’s caregivers. For example, if caregivers were not available for or responsive to the child’s needs, the child will perceive himself or herself as unworthy of being loved, and this negative vision will structure the child’s expectations of the future [37,38,39]. On the contrary, when parents represent a secure base [40], the adolescent will be able to lean on it, which will help the adolescent to imagine his or her present, past and future in much more optimistic and hopeful terms, favoring better psychological adaptation and a better ability to have trusting and supportive peer relationships [41,42].

In particular, adolescence is the period of differentiation from one’s caregivers in favor of peer relationships [43,44,45], although caregivers remain an important internal and external reference point [46] through the indirect influence that they have on one’s beliefs about appropriate social behaviors and relationship models based on attachment experiences [47]. In this regard [48], argued that when social information is likely to cause psychological pain, insecure individuals will be more likely than confident ones to exclude or defensively suppress this information from further processing, because insecure individuals are less likely to have had experiences with an attachment figure in which their painful emotions were understood and elaborated. This will also influence the level of “theory of the mind”, defined as the ability to interpret others’ behaviors within a mentalistic structure in order to understand how oneself and others think, feel, perceive, imagine, react, attribute, infer and so on [49].

Finally, in addition to relationships, attachment also influences coping strategies aimed at dealing with stressful situations [50,51]. Taken together, recent research suggests that successful coping has important implications for the severity of gambling among young people.

The results also revealed that heavier players used more maladaptive forms of coping than others, whether oriented toward emotions or distraction [52,53]. This evidence fits well within [54,55], which suggests that pathological gamblers exhibit various psychological vulnerabilities that leave them ill-equipped, compared to others, to cope with stress. In this context, gambling, akin to other addictive behaviors, is aimed at negotiating negative or stressful experiences when the subject is lacking the resources to find more adequate answers [56].

On these theoretical bases, the present study aims to analyze a hypothesized mediation model exploring both the direct and indirect effects of insecure attachment on gambling disorder by investigating the roles of the developmental perspective, theory of mind (friend) and adaptive responses in that relationship.

## 2. Method

### 2.1. Participants and Procedure

The sample consisted of 178 adolescents (42.1% male and 57.9% female), with a mean age of 17.51 years (*SD* = 0.818), ranging from 16 to 22. The participants were recruited from several secondary schools in Rome. The interview and questionnaire were administered in person in a one-to-one setting by one of the researchers.

Informed consent was obtained from both adolescents and their parents prior to participation in the study. The subjects did not receive any form of payment for participating and were free to leave the study at any time.

### 2.2. Measures

South Oaks Gambling Screen—Revised for Adolescents (SOGS-RA).

The *South Oaks Gambling Screen—Revised for Adolescents* (SOGS-RA; [57]) is a self-report questionnaire used to assess gambling behaviors and gambling-related problems in adolescents. It is made up of 12 dichotomously scored items and other unscored ones investigating the frequency of participation in different gambling activities, the largest amount of money gambled in a day, and parental involvement in problematic gambling.

The SOGS-RA scale identifies three categories: nonproblem gambler (score of 0 or 1), at-risk gambler (score of 2 or 3) and problem gambler (score of 4 or more). For the present study, the Italian version [58] was used. The SOGS demonstrated high internal consistency, with a Cronbach’s alpha coefficient of 0.84.

### 2.3. Friends and Family Interview (FFI)

The *Friends and Family Interview* (FFI; [59,60]) is a semistructured interview designed to assess the attachment representations of adolescents, focusing on oneself, peers (one’s best friend), siblings and parents. It lasts around 45 min and consists of 27 questions about the adolescents and their most significant relationships, with scores ranging from 1 (*“no evidence”*) to 4 (*“marked evidence”*) and including half-points.

The FFI coding system comprises both attachment classifications (*secure-autonomous, insecure-dismissing, insecure-preoccupied* and *insecure-disorganized*) and dimensional scores across numerous domains: (1) Firstly, the coherence of answers is evaluated based on the entire interview, based on their *truth* (based on the presence of convincing evidence), *economy* (based on the amount of given information), *relation* (based on the relevance of the examples provided) and *manner* (based on the maintenance of age-appropriate levels of attention, politeness and interest). (2) Another domain concerns reflective functioning (RF), which includes one’s *developmental perspective* (the ability to relate one’s own present views, feeling and thoughts with past and future attitudes), *theory of mind* (the ability to assume others’ mental perspectives), *diversity of feelings* (the ability to discuss negative and positive affections that could be linked to oneself and significant relationships) and *internal working models* (the availability of a secure base from the subjects’ mothers and fathers, emerging from their narrative). (3) An evaluation of the child’s self-esteem is also given, comprising *social competence*, *school competence* and *self-regard*. (4) Peer relations are explored, in terms of both *frequency of contact* and *quality* of one’s best friendship. (5) Sibling relations are investigated in terms of *warmth*, *hostility* and *rivalry*. (6) The FFI captures affective regulation strategies, in terms of both *defensive response* (idealization, role reversal, anger and derogation) and *adaptive response* to distress. (7) Finally, the differentiation of parental representation is examined by observing the participant’s ability to compare and contrast the quality of one’s relationships with each caregiver. For the present study, the Italian version by [61] was used.

### 2.4. Data Analysis

All of the data analyses were performed with the SPSS statistical software (IBM-SPSS version 25.0, IBM, Armonk, NY, USA) for Windows. Descriptive statistics were calculated. Pearson’s *r* correlations were used to investigate the associations between the variables. Then, the SPSS macroprogram PROCESS 3.4 [62] was used to verify the hypnotized multiple-mediation model. Bootstrapping with 5000 samples and a 95% confidence interval was performed to test the significance of the indirect effect.

## 3. Results

Table 1 shows the descriptive statistics for both the sample and the measures.

The association patterns between the SOGS scores, FFI attachment classifications and other FFI domains are presented in Table 2.

**Figure 1:**
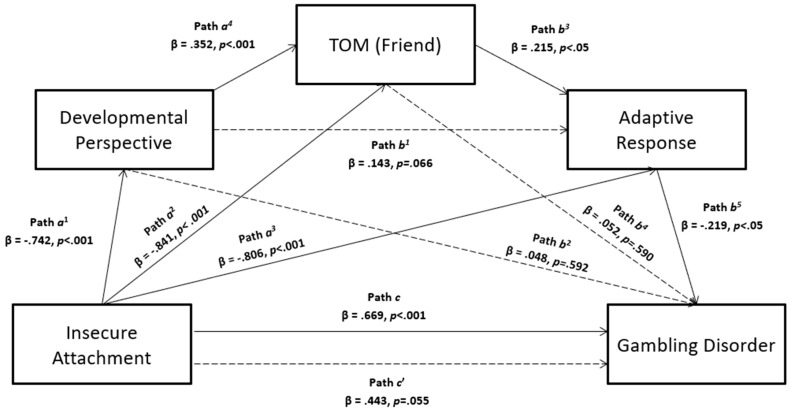
Chained multiple mediation model from insecure attachment to gambling disorder, through developmental perspective, theory of mind (friend) and adaptive response.

The mediation analysis was conducted following [62] procedure, using Model 6 (see Figure 1).

The results showed a significant association between insecure attachment and gambling disorder (*β* = 0.669, *p* < 0.001) when estimating path *c* in Figure 1. The findings also highlighted a significant chained mediation model in which insecure attachment negatively influenced the developmental perspective (path *a^1^* in Figure 1; *β* = −0.742, *p* < 0.001), which affected the theory of mind toward one’s own best friend (path *a^4^* in Figure 1; *β* = 0.352, *p* < 0.001), which in turn predicted the adaptive response to distress (path *b^3^* in Figure 1; *β* = 0.215, *p* < 0.05), which ultimately impacted gambling disease levels (path *b^5^* in Figure 1; *β* = −0.219, *p* < 0.05). However, this finding did not suffer any direct effects from the first two mediators (path *b^2^* in Figure 1 with *β* = 0.048, *p* = 0.592 and path *b^4^* in Figure 1 with *β* = 0.052, *p* = 0.590, respectively). Insecure attachment also negatively and significantly predicted the theory of mind toward one’s best friend (path *a*^2^ in Figure 1; *β* = −0.841, *p* < 0.001) and adaptive response (path *a^3^* in Figure 1; *β* = −0.806, *p* < 0.001), although its direct effect on gambling disorder was not significant (path *c’* in Figure 1; *β* = 0.443, *p* = 0.055), indicating a complete mediation after controlling the mediators (*R*^2^= 0.115, F_4,148_= 4.825, *p=* 0.001) (see Table 3).

The bootstrapping procedure confirmed the statistical stability of this chained mediation model and the significance of its indirect effect (boot LLCI = 0.001, boot ULCI = 0.063; see Table 4).

## 4. Discussion

Pathological gambling is a multifaceted phenomenon with numerous underlying factors in its development and maintenance [29,63]. On the other hand, adolescence is an extremely vulnerable phase [64], during which subjects are more inclined to be involved in risky behaviors [65]. Therefore, the early onset of this disorder can have a potentially devastating effect on the individual’s development, which is strongly associated with a more serious and chronic course of the disease as well as with various comorbidities [66,67]. On this basis, we investigated the impacts of insecure attachment on gambling disorder in adolescence and also analyzed the mediating roles of developmental perspective, theory of mind (friend) and adaptive response to distress.

Consistent with field research [17,18,19], the results highlighted a significant role of insecure attachment in predicting the symptomatic expression of gambling in adolescents. However, our data also showed that this association was achieved through an indirect path, by influencing some core aspects in the adolescent’s adjustment: insecure attachment negatively impacted the development perspective, theory of mind toward one’s best friend and adaptive response to stress, which were linked to each other by a sequential influence. This is in line with evidence considering secure attachment as the starting point for the construction of a functional time perspective, a theory of mind and emotional regulation that will allow for adequate and adaptive self-development [68,69,70,71]. By contrast, chronically negative early relationships with caregivers are a risk factor for opposite effects, which could lead to psychopathology [50,72,73,74].

Moreover, the domination of several time categories may be responsible for limited psychosocial functioning [75,76], and adolescents who focus on the present and on the immediate future have a greater risk of engaging in high-risk behaviors such as substance abuse [76,77,78]. Therefore, our results showed that a poor developmental self-vision predicted a dysfunctional theory of mind toward one’s best friend. This could hinder the formation of positive peer relationships, which are crucial for the development of one’s identity [79,80]. The ability to interpret others’ behaviors within a mentalistic structure to understand how oneself and others think, feel, perceive, imagine, react, attribute and infer [49] influences adaptation strategies for social interactions [45,72]. When relationships with peers are negative and problematic, an adolescent may experience dysfunctional responses such as delinquent and aggressive behaviors [12], symptoms of depression [7], increased risk of comorbidity [81], general health problems [10] and gambling disorders [8]. All of these factors provide an understandable explanation for the connection highlighted by the data between one’s theory of the mind (friend) and adaptive responses to stress, which in turn affect gambling behaviors. Indeed, according to previous research [50,52,53,54,55,56], gambling disorder in adolescence, as with other addictive behaviors, could be interpreted as a dysfunctional response used to cope with stress and negative situations when the subject lacks the resources to find more adequate answers. Adolescents with insecure attachment tend to have maladaptive emotion-regulation strategies [20]. Based on this perspective, pathological gambling could be an attempt at self-medication [23,24].

## 5. Conclusions

Our study adds two main aspects. First, a multiple-mediation model was used to explore some latent psychological constructs in the pathological manifestation of gambling, specifically insecure attachment, deficits in the developmental perspective, a failed theory of the mind, and nonadaptive responses. Second, we used the Friends and Family Interview (FFI; [59,60]), a semistructured interview similar to the Adult Attachment Interview (AAI; [82]), which detects attachment representations among adolescents. Compared to the AAI, the FFI is focused on oneself and one’s peers (best friend), siblings and parents, and it systematically investigates the adolescent’s perspective, instead of comparing one’s semantic and episodic memories of past experiences with attachment figures as the AAI does. Our results can be applied to psychological interventions based on restructuring attachment patterns, developing theory of mind and reflective self-functioning, promoting adaptive coping strategies, and improving relationships with peers.

Importantly, this study has some limitations. Its cross-sectional nature limited the possibility of establishing inferences about the causal/directional relationships between the variables. Future longitudinal research may be important to consolidate the conclusions drawn from this study and to investigate results also in adult pathological gamblers. Additionally, gambling behaviors were analyzed using a self-report measure, which, although quick and easy to administer, exposes participants to the risk of bias such as social desirability biases. The measure’s integration with different methods (e.g., interviews) could be useful in future studies. Finally, the different subtypes of gambling were not analyzed. The exploration of such data could be an important challenge for future research to delineate different profiles of pathological gamblers.

## Figures and Tables

**Table 1:** Descriptive statistics.

	Means (SD)					
	Total (*N* = 178)	Gender		Gambling Disease		
		Boys (*N* = 75)	Girls (*N* = 103)	Absent (*N* = 141)	Risk (*N* = 24)	Pathological (*N* = 13)
Age	17.51 (0.82)	17.52 (0.88)	17.50 (0.78)	17.38 (0.651)	18.12 (1.30)	17.77 (0.83)
Measures						
SOGS	0.95 (1.81)	1.71 (2.41)	0.40 (0.88)	0.20 (0.40)	2.58 (0.50)	6.08 (2.40)
FFI						
Attachment Patterns						
Secure-autonomous	2.74 (0.81)	2.62 (0.84)	2.84 (0.76)	2.90 (0.73)	2.10 (0.83)	2.36 (0.81)
Insecure-dismissing	1.95 (0.88)	2.09 (0.92)	1.83 (0.84)	1.80 (0.79)	2.62 (0.97)	2.17 (1.03)
Insecure-preoccupied	1.45 (0.67)	1.46 (0.76)	1.44 (0.60)	1.43 (0.63)	1.50 (0.72)	1.58 (1.00)
Disorganized	1.26 (0.63)	1.24 (0.65)	1.27 (0.63)	1.23 (0.59)	1.33 (0.82)	1.46 (0.67)
Coherence						
Truth	2.87 (0.72)	2.81 (0.76)	2.92 (0.68)	2.98 (0.69)	2.39 (0.58)	2.69 (0.86)
Economy	2.79 (0.65)	2.71 (0.71)	2.84 (0.61)	2.91 (0.61)	2.33 (0.57)	2.38 (0.77)
Relation	2.69 (0.80)	2.53 (0.85)	2.80 (0.73)	2.82 (0.76)	2.17 (0.72)	2.31 (0.75)
Manner	3.25 (0.73)	3.20 (0.69)	3.29 (0.76)	3.36 (0.69)	2.79 (0.83)	3.00 (0.58)
Overall coherence	2.74 (0.76)	2.62 (0.88)	2.83 (0.67)	2.85 (0.65)	2.74 (1.10)	2.75 (0.50)
Reflective Functioning						
Developmental perspective	2.77 (0.93)	2.74 (0.89)	2.79 (0.95)	2.85 (0.91)	2.35 (0.86)	2.73 (1.01)
Theory of mind						
Mother	2.68 (0.80)	2.61 (0.84)	2.73 (0.77)	2.75 (0.76)	2.48 (0.85)	2.31 (1.03)
Father	2.58 (0.83)	2.31 (0.87)	2.60 (0.78)	2.54 (0.82)	2.26 (0.81)	2.23 (0.93)
Friend	2.43 (0.91)	2.26 (0.90)	2.55 (0.90)	2.54 (0.89)	1.96 (0.88)	2.23 (0.93)
Sibling	2.42 (0.84)	2.27 (0.88)	2.52 (0.80)	2.57 (0.77)	1.82 (0.88)	1.78 (0.83)
Teacher	2.59 (0.75)	2.58 (0.66)	2.60 (0.80)	2.64 (0.73)	2.25 (0.91)	2.69 (0.48)
Diversity of feelings						
Self	2.73 (0.97)	2.68 (0.95)	2.76 (0.99)	2.83 (0.99)	2.38 (0.82)	2.36 (0.81)
Mother	2.48 (1.06)	2.37 (1.04)	2.56 (1.07)	2.60 (1.04)	2.09 (1.04)	2.00 (1.04)
Father	2.54 (0.80)	2.51 (0.76)	2.56 (0.84)	2.60 (0.81)	2.43 (0.79)	2.17 (0.72)
Friend	2.46 (0.93)	2.33 (0.87)	2.54 (0.96)	2.53 (0.96)	2.08 (0.78)	2.46 (0.78)
Sibling	2.60 (0.83)	2.56 (0.78)	2.62 (0.88)	2.72 (0.79)	2.12 (0.89)	2.20 (0.79)
Secure base/safe haven						
Mother	2.52 (0.83)	2.38 (0.93)	2.63 (0.73)	2.60 (0.80)	2.23 (0.92)	2.25 (0.87)
Father	2.17 (0.73)	2.13 (70)	2.20 (0.76)	2.21 (0.72)	1.96 (0.81)	2.15 (0.69)
Self-esteem						
Social competence	2.86 (68)	2.79 (0.72)	2.92 (0.64)	2.56 (0.53)	2.54 (0.72)	2.77 (0.83)
School competence	2.90 (0.57)	2.81 (0.60)	2.97 (0.54)	2.93 (0.64)	2.71 (0.55)	3.00 (0.95)
Self-regard	2.61 (0.67)	2.67 (0.61)	2.57 (0.69)	2.67 (0.64)	2.30 (0.77)	0.62 (0.51)
Friend relationship						
Frequency of contact	2.63 (1.01)	2.66 (1.03)	2.61 (0.99)	2.69 (0.99)	2.70 (0.97)	1.92 (1.04)
Quality of relation	2.77 (0.78)	2.65 (0.82)	2.85 (0.73)	2.85 (0.75)	2.46 (0.78)	2.54 (0.88)
Sibling relationship						
Warmth	2.83 (0.82)	2.67 (0.84)	2.94 (0.80)	2.93 (0.80)	2.29 (0.77)	2.60 (0.84)
Hostility	1.41 (0.64)	1.54 (0.72)	1.33 (0.57)	1.36 (0.62)	1.56 (0.63)	1.80 (0.79)
Rivalry	1.13 (0.33)	1.15 (0.37)	1.11 (0.32)	1.11(0.32)	1.12 (0.33)	1.30 (0.48)
Affective regulation						
Idealization						
Self	1.19 (0.43)	1.20 (0.44)	1.19 (0.42)	1.17 (0.40)	1.29 (0.55)	1.23 (0.44)
Mother	1.76 (0.72)	1.83 (0.77)	1.72 (0.68)	1.70 (0.64)	2.09 (1.00)	1.83 (0.84)
Father	1.68 (0.68)	1.64 (0.70)	1.71 (0.67)	1.65 (0.65)	1.83 (0.89)	1.69 (0.63)
Role reversal						
Mother	1.25 (0.50)	1.29 (0.52)	1.22 (0.50)	1.22 (0.50)	1.35 (0.49)	1.38 (0.51)
Father	1.15 (0.42)	1.15 (0.44)	1.15 (0.41)	1.14 (0.39)	1.29 (0.62)	1.00 (0.00)
Anger						
Mother	1.24 (0.52)	1.30 (0.57)	1.19 (0.47)	1.23 (0.51)	1.38 (0.65)	1.08 (0.28)
Father	1.19 (0.44)	1.31 (0.55)	1.11 (0.31)	1.17 (0.38)	1.25 (0.53)	1.31 (0.75)
Derogation						
Self	1.17 (0.44)	1.21 (0.45)	1.14 (0.43)	1.18 (0.42)	1.08 (0.41)	1.23 (0.60)
Mother	1.10 (0.41)	1.17 (0.45)	1.05 (0.37)	1.06 (0.37)	1.21 (0.51)	1.31 (0.48)
Father	1.12 (0.37)	1.16 (0.44)	1.10 (0.30)	1.09 (0.32)	1.13 (0.34)	1.42 (0.67)
Adaptive Response	2.69 (0.80)	2.56 (0.84)	2.78 (0.76)	2.83 (0.77)	2.17 (0.72)	2.23 (0.73)
Differentiation of parental	3.05 (0.73)	3.04 (0.75)	3.06 (0.71)	3.15 (0.67)	2.67 (0.96)	2.85 (0.56)
representations						

**Table 2:** Correlation matrix between South Oaks Gambling Screen (SOGS), Friends and Family Interview (FFI) attachment patterns and FFI domains.

	FFI Attachment Patterns					SOGS
	4-Way				2-Way	
	Secure-Autonomous	Insecure-Dismissing	Insecure-Preoccupied	Disorganized-Disoriented	Secure/Insecure	
SOGS	−0.263 **	0.186 *	0.046	0.051	0.311 **	1
FFI						
Coeherence						
Truth	0.791 **	−0.658 **	0.002	−0.378 **	−0.592 **	−0.156 *
Economy	0.728 **	−0.520 **	−0.134	−0.337 **	−0.592 **	−0.249 **
Relation	0.752 **	−0.482 **	−0.138	−0.351 **	−0.567 **	−0.229 **
Manner	0.578 **	−0.332 **	−0.197 *	−0.236 **	−0.396 **	−0.182 *
Overall coherence	0.733 **	−0.554 **	−0.075	−0.402 **	−0.530 **	−0.157
Reflective Functioning						
Developmental perspective	0.515 **	−0.499 **	0.115	−0.207 **	−0.320 **	−0.076
Theory of mind						
Mother	0.488 **	−0.381 **	0.079	−0.156 *	−0.249 **	−0.132
Father	0.442 **	−0.365 **	0.127	−0.153	−0.286 **	−0.126
Friend	0.514 **	−0.522 **	0.033	−0.224 **	−0.480 **	−0.148
Sibling	0.377 **	−0.391 **	−0.108	−0.198 *	−0.371 **	−0.264 **
Teacher	0.415 **	−0.452 **	0.124	−0.152	−0.303 **	−0.035
Diversity of feelings						
Self	0.572 **	−0.536 **	0.067	−0.351 **	−0.429 **	−0.126
Mother	0.559 **	−0.459 **	0.129	−0.192 *	−0.390 **	−0.210 **
Father	0.520 **	−0.457 **	0.135	−0.059	−0.344 **	−0.149
Friend	−0.077	0.100	−0.055	−0.033	0.132	−0.044
Sibling	0.656 **	−0.538 **	−0.060	−0.324 **	−0.604 **	−0.186 *
Secure base/safe haven						
Mother	0.612 **	−0.402 **	−0.165 *	−0.261 **	−0.503 **	−0.123
Father	0.468 **	−0.345 **	−0.037	−0.201 *	−0.375 **	−0.077
Self-esteem						
Social competence	0.462 **	−0.411 **	−0.053	−0.296 **	−0.320 **	−0.100
School competence	0.374 **	−0.382 **	−0.040	−0.255 **	−0.343 **	0.003
Self-regard	0.423 **	−0.317 **	−0.074	−0.289 **	−0.304 **	−0.115
Friend relationship						
Frequency of contact	0.141	−0.220 **	0.037	−0.162 *	−0.140	−0.123
Quality of relation	0.585 **	−0.561 **	0.087	−0.248 **	−0.458 **	−0.107
Sibling relationship						
Warmth	0.398 **	−0.340 **	−0.034	−0.283 **	−0.333 **	−0.157
Hostility	−0.227 *	0.109	0.041	0.035	0.159	0.143
Rivalry	−0.043	−0.068	0.146	−0.013	0.049	0.143
Affective regulation						
Self	−0.135	0.183 *	−0.043	0.173 *	0.120	0.038
Mother	−0.429 **	0.574 **	−0.246 **	0.176 *	0.373 **	0.035
Father	−0.388 **	0.439 **	−0.205 **	0.154 *	0.342 **	−0.013
Role reversal						
Mother	−0.036	−0.201 *	0.263 **	−0.011	−0.014	0.124
Father	−0.030	0.019	0.160 *	0.216 **	0.049	−0.055
Anger						
Mother	−0.202 *	−0.055	0.328 **	0.150	0.209 **	0.003
Father	−0.118	−0.151	0.364 **	0.020	0.085	0.133
Derogation						
Self	−0.250 **	0.136	0.115	0.222 **	0.182 *	0.022
Mother	−0.360 **	0.212 **	0.122	0.084	0.326 **	0.136
Father	−0.208 *	−0.026	0.309 **	0.018	0.187 *	0.228 **
Adaptive Response	0.635 **	−0.391 **	−0.134	−0.261 **	−0.505 **	−0.290 **
Differentiation of parental representations	0.335 **	−0.441 **	0.210 **	−0.045	−0.312 **	−0.243 **

** Correlation is significant at the 0.01 level (2-tailed). * Correlation is significant at the 0.05 level (2-tailed).

**Table 3:** Mediation model coefficients.

Antecedent	Consequent															
		M1				M2				M3				Y		
		Coeff.	SE	*p*		Coeff.	SE	*p*		Coeff.	SE	*p*		Coeff.	SE	*p*
X	*a* ^1^	−0.691	0.167	<0.001	*a* ^2^	−0.760	0.147	<0.001	*a^3^*	−0.653	0.146	<0.001	*c’*	0.930	0.401	0.022
M1		-	-	-	*a* ^4^	0.341	0.068	<0.001	*b^1^*	0.124	0.067	0.066	*b* ^2^	0.094	0.175	0.592
M2		-	-	-		-	-	-	*b^3^*	0.193	0.075	0.011	*b* ^4^	0.106	0.196	0.590
M3		-	-	-		-	-	-		-	-	-	*b* ^5^	−0.494	0.211	0.021
Constant	*i_M__1_*	3.631	0.220	<0.001	*i_M2_*	2.456	0.308	<0.001	*i_M2_*	2.698	0.335	<0.001	*i_Y_*	0.616	1.034	0.545
		*R*^2^= 0.102 *F*(1,151)= 17.064, *p* <0.001				*R*^2^= 0.336 *F*(2,150)= 37.863, *p* < 0.001				*R*^2^= 0.317 *F*(3,149)= 0.317, *p* < 0.001				*R*^2^= 0.115 *F*(4,148)= 4.825, *p* = 0.001		

Note: X = Insecure attachment; M1 = developmental perspective; M2 = theory of mind (friend); M3 = adaptive response; Y = gambling disorder.

**Table 4:** Model effect indices.

Total Effect	Direct Effect	Indirect Effect	Partial Standardized Indirect Effect	Bootstrapping 95% CI
1.22	0.93	0.02	0.01	(0.001, 0.063]
